# 

**Published:** 1887-12-17

**Authors:** 


### CONTENTS.

**London's Churches, Chapels, and People** - 189

**New Sources of Income for the Hospitals.** By the Duke of Argyll - 191

**London's Great Problem Solved** - 193

**The Doctor's Pulpit.**—The Spirit Bottle - 197

**The Hydrobromates** - 198

**Clinical and Therapeutic Notes.** By C. R. Illingworth, M.D., M.R.C.S.—Pyrexia and Anti-pyretics - 199

**Notes and News** - 200

**Annotations.**—A Merry Christmas.—The Compliments of the Season.—£200 a-year for a Hospital Physician - 203

**The National Pension Fund for Nurses and Hospital Officials** - 204

**The Students' Column** - 204

**The Registration of Qualified Nurses.** By J. C. Steele, M.D., Superintendent of Guy's Hospital - 205

**East and West.** By Leslie Keith. Chapter XII.—David's Hour of Triumph - 207

**A County Hospital.** By Our Special Commissioner - 209

**Common Accidents of Every-day Life.** By R. A. P. Forrester, B.A.Dub.—Accident to a Child in a Perambulator - 210

**Amusements for Convalescents.**—Palmistry Notes by A Lady - 211

**Amusements with Profit for all Ages.**—Competitions, Puzzles, Children's Corner, etc. - 212

The average issue of "The Hospital" now exceeds **10,000 COPIES WEEKLY**, and the circulation is steadily increasing week by week.

